# A Readability Analysis of Online Cardiovascular Disease-Related Health Education Materials

**DOI:** 10.3928/24748307-20190306-03

**Published:** 2019-04-10

**Authors:** Varun Ayyaswami, Divya Padmanabhan, Manthan Patel, Arpan Vaikunth Prabhu, David R. Hansberry, Nitin Agarwal, Jared W. Magnani

## Abstract

**Background:**

Online cardiovascular health materials are easily accessible with an Internet connection, but the readability of its content may limit practical use by patients.

**Objective:**

The goal of our study was to assess the readability of the most commonly searched Internet health education materials for cardiovascular diseases accessed via Google.

**Methods:**

We selected 20 commonly searched cardiovascular disease terms: aneurysm, angina, atherosclerosis, cardiomyopathy, congenital heart disease, coronary artery disease, deep vein thrombosis, heart attack, heart failure, high blood pressure, pericardial disease, peripheral arterial disease, rheumatic heart disease, stroke, sudden death, valvular heart disease, mini-stroke, lower extremity edema, pulmonary embolism, and exertional dyspnea. Terms were selected on Google and selected up to 10 results in order of presentation in the search results by reviewing a maximum of 15 pages of Google search results specifically providing education toward patients to yield 196 total patient education articles.

**Key Results:**

All readability measures assessing grade level measures found the 196 articles were written at a mean 10.9 (*SD* = 1.8) grade reading level. Moreover, 99.5% of the articles were written beyond the 5th- to 6th-grade level recommended by the American Medical Association.

**Conclusions:**

Given the prominent use of online patient education material, we consider readability as a quality metric that should be evaluated prior to online publication of any health education materials. Further study of how to improve the readability of online materials may enhance patient education, engagement, and health outcomes. **[*HLRP: Health Literacy Research and Practice*. 2019;3(2):e74–e80.]**

**Plain Language Summary:**

Patients often use Google as a tool for understanding their medical conditions. This study examined the readability of articles accessed via Google for commonly searched cardiovascular diseases and found all articles were written above reading grade levels appropriate for patients. We hope this study will promote the importance of ensuring that online patient education articles are written at appropriate reading levels.

Approximately 52 million adults in the United States seek health information online, and 70% of them report that the Internet influences their decision-making about treatments (Rainie & Susannah, 2000). Although online health materials are easily accessible with an Internet connection, the readability of their content may limit practical use by patients (Agarwal, Hansberry, & Prabhu, 2017). Health literacy is a widely prevalent challenge, and the mean reading level of adults in the U.S. is estimated as 7th- to 8th-grade level (Kutner, Greenberg, Jin, & Paulsen, 2006; National Assessment of Adult Literacy, 2003). Thus, the American Medical Association (AMA) and National Work Group on Cancer and Health have recommended that educational materials for patients be on a 5th- to 6th-grade reading level (Cotugna, Vickery, & Carpenter-Haefele, 2005; Weiss, 2007). Unfortunately, previous studies performed by our group and others show that Internet-based patient educational materials do not follow these recommendations (Bernard, Cooke, Cole, Hachani, & Bernard, 2018; Hansberry, Kraus, Agarwal, Baker, & Gonzales, 2014; Hansberry, Ramchand, et al., 2014; Kher, Johnson, & Griffith, 2017; Lee, Berg, Jazayeri, Chuang, & Eisig, 2019; Prabhu, Hansberry, Agarwal, Clump, & Heron, 2016).

Cardiovascular diseases have complex mechanisms and etiologies that are difficult for patients to comprehend. Limited health literacy is a barrier to the successful clinical management of patients with cardiovascular disease and is associated with higher cardiovascular disease risk scores (Magnani et al., 2018; Van Schaik et al., 2017) and higher rates of all-cause mortality in patients with heart failure (Peterson et al., 2011). Previous studies have shown that printed educational materials for cardiovascular disease patients are not appropriate for patients with limited health literacy (Eames, McKenna, Worrall, & Read, 2003; Hill-Briggs & Smith, 2008). Furthermore, cardiovascular professional societies have promoted online educational materials that exceed national recommendations (Kapoor, George, Evans, Miller, & Liu, 2017). Thus, the goal of our study was to assess the readability of commonly accessed Internet health education materials for cardiovascular diseases accessed via Google. We hypothesized that the readability of patient-specific health education materials available online through Google are written well above nationally recommended reading levels.

## Methods

We selected 20 patient-oriented terms related to cardiovascular disease for our study based on our clinical experience using the World Health Organizations broad definition of cardiovascular disease as a guideline for term selection (https://www.who.int/news-room/fact-sheets/detail/cardiovascular-diseases-(cvds). Terms selected were aneurysm, angina, atherosclerosis, cardiomyopathy, congenital heart disease, coronary artery disease, deep vein thrombosis, heart attack, heart failure, high blood pressure, pericardial disease, peripheral arterial disease, rheumatic heart disease, stroke, sudden death, valvular heart disease, mini-stroke, lower extremity edema, pulmonary embolism, and exertional dyspnea.

We entered these terms separately into Google and selected up to 10 results in order of presentation in the search results by reviewing a maximum of 15 pages of Google search results specifically providing education toward patients to yield 196 total patient education articles. A search result was considered written for patient education unless the article was explicitly indicated for a medical professional. We reformatted articles to plain text in Microsoft Word and deleted material unrelated to patient education. This included removal of figures and their legends, disclaimers, copyright notices, acknowledgments, multimedia, captions, author information, web page navigation text, and references. We quantitatively evaluated the readability according to 10 readability measures (to minimize influence of any individual scale) with commercially available software (Readability Studio; Professional Edition Version 2012.1, Oleander Software, Ltd, Vandalia, OH). These readability measures included the Flesch Reading Ease (FRE), Coleman-Liau Index (CLI), Flesch-Kincaid Grade Level (FKGL), Gunning Fog Index (GFI), FORCAST Readability Formula, New Dale-Chall formula (NDC), New Fog Count (NFC), Simple Measure of Gobbledygook (SMOG) Index, Fry Readability Formula (FRF), and Raygor Readability Estimate (RRE). The FRE test assesses readability through word, syllable, and sentence counts with lower scores indicating more difficult text (0–29, *very difficult*; 30–49, *difficult*; 50–59, *fairly difficult*; 60–69, *standard*; 70–79, *fairly easy*; 80–89, *easy*; 90–100, *very easy*) (Flesch, 1948; Jindal & MacDermid, 2017). The nine other readability measures provide grade level values with higher values implicating more complex text. The CLI measures readability by analyzing the number of letters and sentences per 100 words (Coleman & Liau, 1975). FKGL analyzes the average number of words per sentence and average number of syllables per word (Kincaid, Fishburne, Rogers, & Chissom, 1975). The GFI uses the number of sentences, number of words with three or more syllables, and the average sentence length (Gunning, 1952). The FORCAST Readability Formula evaluates the readability of a material on the number of single-syllable words present in a 150-word section (Caylor, Sticht, Fox, & Ford, 1973). NDC uses a count of hard words and the number of words per sentence (Chall & Dale, 1995). NFC assesses readability by counting the number of sentences, complex words, and easy words (Kincaid et al., 1975). The SMOG Index evaluates readability through the number of polysyllabic words and the number of sentences (Caylor et al., 1973). The FRF examines the average number of sentences and syllables in every 100 words (Fry, 1968). Finally, the RRE determines readability grade-level based on the mean number of sentences and number of words with 6 or more letters (Raygor, 1977). Institutional review board approval was not required because all data were publicly available online.

## Results

**Figure 1.**
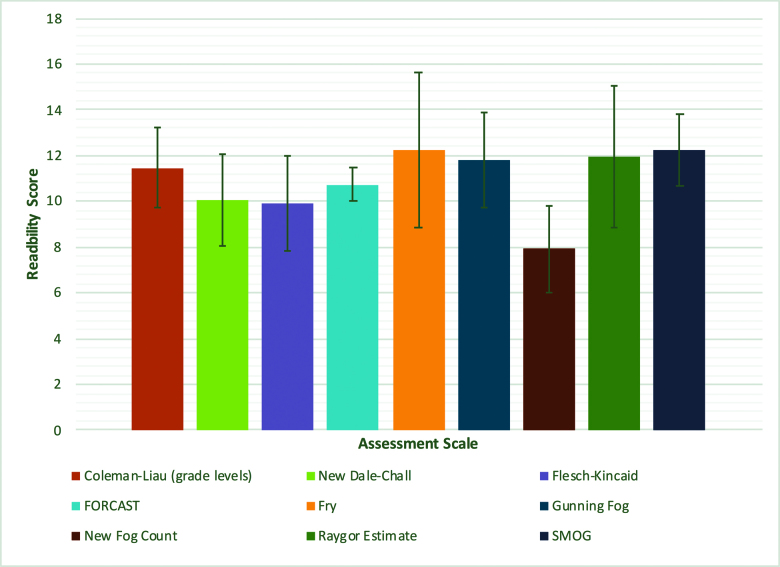
The mean readability score for 196 collected articles yielded from commonly searched cardiovascular diseases on Google for readability analysis as measured by nine established readability scales that assess grade level. Scores correspond to the corresponding academic grade level required for reading. Error bars refer to standard deviation. All readability scales determined the mean reading level of the 196 collected articles that were above the grade-level recommendations promoted by the American Medical Association.

**Figure 2.**
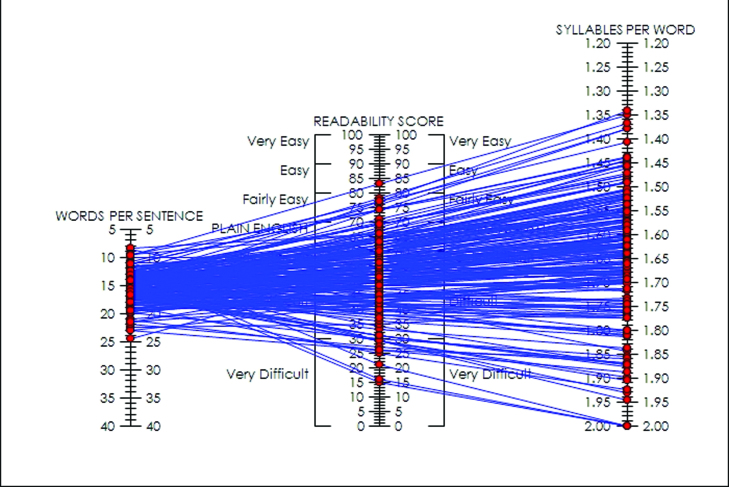
The Flesch Reading Ease scores for 196 collected articles yielded from commonly searched cardiovascular diseases on Google for readability analysis. This test evaluates readability through syllable count and sentence length to calculate a score between 0 and 100 to indicate readability ease, with lower scores indicating greater difficulty. The Flesch Reading Ease mean score of the 196 analyzed articles (52.3 ± 12.1) corresponded to a qualitative readability score of *fairly difficult* to read. The figure plots the qualitative and quantitative scores of the 196 articles analyzed.

**Figure 3.**
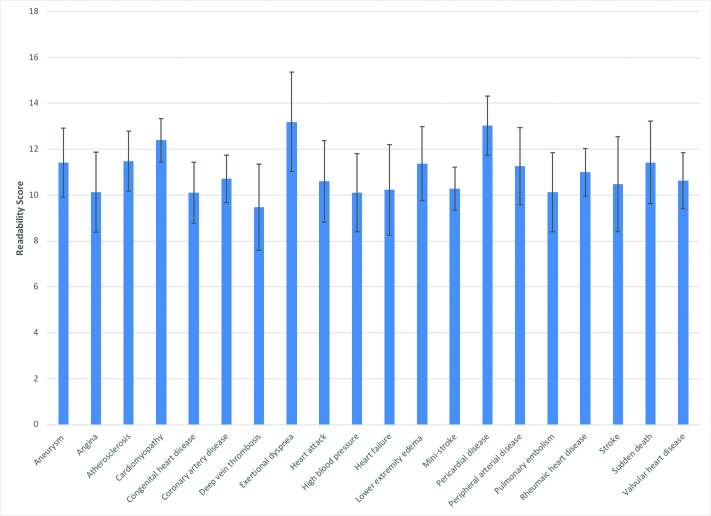
The mean readability score for commonly searched cardiovascular diseases on Google as measured by nine established readability scales that estimate grade level. Scores correspond to the academic grade level required for reading. Error bars refer to standard deviation. Readability scales determined the mean reading level for the 20 terms that were above the grade-level recommendations promoted by the American Medical Association.

The nine readability measures assessing grade level found that the 196 articles were written at a mean 10.9 (standard deviation [*SD*] = 1.8) grade reading level (**Figure 1**). A single article, 1 of 196 articles, was written at the recommended 5th- to 6th-grade level. The FRE test identified the articles as being “fairly difficult” to read (*M* = 52.3, *SD* = 12.1 of 100) (**Figure 2**). Articles associated with exertional dyspnea had the highest reading grade level (*M* = 13.2, *SD* = 2.2) (**Figure 3**). Articles on deep vein thrombosis had the lowest readability scores with an average grade-level score of 9.5 (*SD* = 1.9) (**Figure 3**). As a whole, the articles associated with each search term were above national recommendations as measured by all nine readability measures assessing grade level.

## Discussion

Our study revealed that the top 10 articles on Google for 20 commonly searched cardiovascular disease terms were written at an average 10.9 (*SD* = 1.8) grade reading level as measured by nine established readability scales that evaluate grade level. Moreover, 99.5% of articles were written beyond the 5th- to 6th-grade level promoted by national organizations for patients (Cotugna et al., 2005; Weiss, 2007). Our results demonstrate a disconnect between frequently searched cardiovascular disease-related health education materials and the reading grade level for patients. The AMA has promoted readability to make health-related educational materials accessible to patients (Weiss, 2007). In the context of both the numerous studies in the medical field that have demonstrated that Internet-based patient educational materials do not follow national readability recommendations (Bernard et al., 2018; Crihalmeanu, Prabhu, Hansberry, Agarwal, & Fine, 2018; Hansberry, Ayyaswami et al., 2017; Hansberry, D'Angelo, et al., 2018; Kher et al., 2017; Lee at al., 2019; Prabhu, Crihalmeanu, et al., 2017; Prabhu, Gupta, et al., 2016; Prabhu, Hansberry, et al., 2016; Prabhu, Kim, et al., 2017; Prabhu et al., 2018) and the promotion of educational materials that exceed readability recommendations by cardiovascular professional societies (Kapoor et al., 2017), the gap that we observed between the readability of online materials and patients' reading level has important implications for patients and health care delivery.

Specifically, limited health literacy has been associated with nonadherence to treatment plans, increased health care costs, and greater hospitalization rates (King, 2010). Health literacy challenges are common among older adults, who have increased risk from multiple chronic and cardiovascular diseases (King, 2010). For example, patients with heart failure with limited health literacy had a significantly higher rate of unplanned health care use in the 30 days after hospital discharge (48.3%) compared to those with adequate health literacy (34.9%) (Cox et al., 2016). Furthermore, there was an increased risk of death after hospitalization for acute heart failure in limited health literacy patient populations (McNaughton et al., 2015). Patients with limited health literacy were more than 2 times as likely to be unaware of their atrial fibrillation diagnosis compared to patients with adequate health literacy (24.6% vs. 11.9%) (Reading et al., 2017). In addition, among adults with hypertension seeking treatment in primary care centers, adequate health literacy is associated with increased medication adherence and lower blood pressure levels (Wannasirikul, Termsirikulchai, Sujirarat, Benjakul, & Tanasugarn, 2016).

We suggest increased readability of online health education materials may exert a protective effect against the negative health outcomes associated with cardiac patients who suffer from limited health literacy. Although not the direct focus of this study, we propose such a link between appropriate online patient education and improved health care delivery and outcomes may be an interesting area of future research. With this in mind, professional societies, academic facilities, and others using the Internet to promote patient education should evaluate the readability of their online materials. Moreover, given the prominent use of such online patient education material (Rainie & Susannah, 2000), we consider readability as a quality metric and specifically encourage the use of commercially available readability software prior to publication of any online heath education materials to ensure appropriate readability. Improving the readability of patient educational materials is low cost, patient-centered, and makes the information more accessible to patients (Agarwal, Hansberry & Prabhu, 2017).

## Study Limitations

An important limitation of our study is that readability alone does not imply the factual accuracy of written education materials. The study also did not address if the patient education materials are comprehensive in their scope or adhere to patient empowerment guidelines to be action oriented. Future studies could evaluate the accuracy, understandability, and actionability by employing assessments such as the Patient Education Materials Assessment Tool (Shoemaker, Wolf, & Brach, 2014). Furthermore, including nontext equivalents such as figures and graphics can help compensate for readability concerns (Agarwal, Hansberry & Prabhu, 2017), but we propose there is still a need for the development of comprehendible written education materials when nontext equivalents cannot adequately communicate health information alone. It is important to note that there are discrepancies in readability grade level recommendations, and we chose to focus on national recommendations presented by the AMA and National Work Group on Cancer and Health. We also acknowledge that our study only provides a broad overview of both common and rare cardiovascular disease term readability. Thus, future studies should use the results of our study to more narrowly analyze patient education materials for individual cardiac disease terms of interest. In future studies, we intend to explore specific terms in more detail by including comparisons by website (e.g., WebMD vs. Mayo Clinic).

## Conclusions

We identified that cardiac-specific online patient education materials commonly accessed through Google exceeds grade-level recommendations promoted by the AMA. Future studies should assess the readability of health materials promoted on newer online platforms, such as mobile applications and social media, and the effect of increased readability on health care delivery and outcomes for patients with limited health literacy. Ultimately, further study of how to improve readability of online patient materials may enhance patient education, engagement, and health outcomes by allowing patients with limited health literacy to take a more active role in their health.
